# P114 The Great Pretender: Prolidase Deficiency Masquerading as Refractory Cutaneous Vasculitis

**DOI:** 10.1093/rap/rkag113.144

**Published:** 2026-11-03

**Authors:** Neeraj Jain, Lalit Duggal, Mansi Setia, Pragya Garg, Eshan Taneja, Naina Mehta

**Affiliations:** Sir Gangaram Hospital, New Delhi, India; Sir Gangaram Hospital, New Delhi, India; Sir Gangaram Hospital, New Delhi, India; Sir Gangaram Hospital, New Delhi, India; Sir Gangaram Hospital, New Delhi, India; Sir Gangaram Hospital, New Delhi, India

## Abstract

**Introduction:**

Chronic ulcerative skin lesions with immune dysregulation present a significant diagnostic challenge due to their overlap with autoimmune diseases, vasculitis, inflammatory dermatoses and monogenic disorders. Early disease onset, atypical clinical features and failure to respond to conventional immunosuppressive therapy should prompt consideration of rare inherited conditions. We report a young woman with recurrent lower-limb ulcers, hypergammaglobulinaemia and autoantibody positivity, in whom genetic evaluation ultimately established the diagnosis of prolidase deficiency.

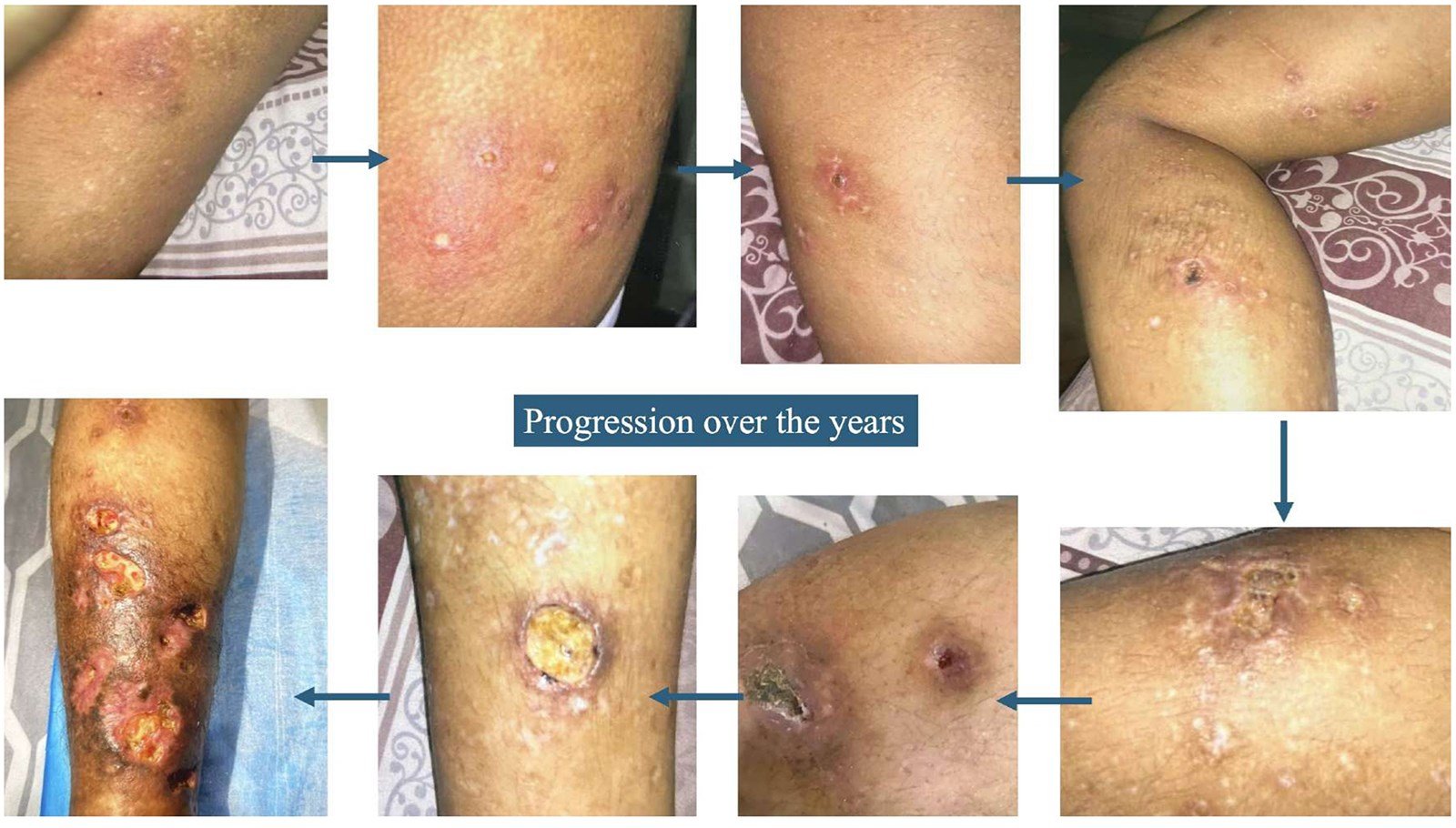

**P114 Figure 1**

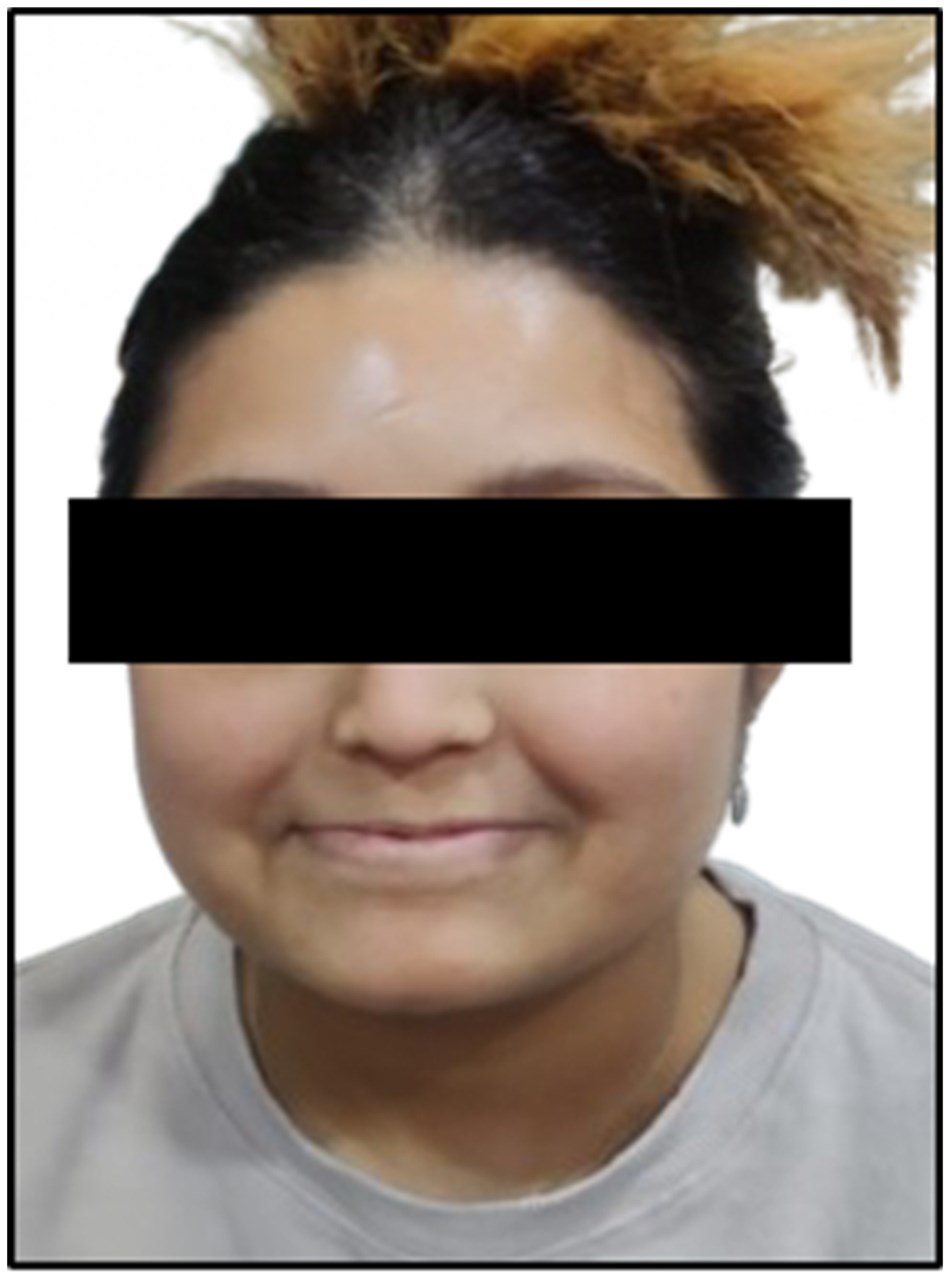

**P114 Figure 2**

**Case description:**

A 20-year-old woman of Indian origin, the only child of non-consanguineous parents and currently residing in Canada, presented with a 14-year history of recurrent painful ulcerative skin lesions predominantly involving the lower limbs. The lesions which started at the age of 6, initially appeared as papules, progressed to ulcers and healed with scarring. She had no symptoms suggestive of systemic vasculitis or connective tissue disease but had a history of learning difficulty. Initial skin biopsy in childhood demonstrated a dense inflammatory infiltrate extending into the subcutis, consistent with pyoderma gangrenosum, and she received antibiotics, corticosteroids and later tofacitinib with limitedbenefit. A repeat biopsy in 2025 suggested livedoid vasculopathy. Given the chronic refractory course, the differential diagnoses included deficiency of adenosine deaminase 2 (DADA2), cutaneous polyarteritis nodosa, connective tissue disease-associated vasculitis, pyoderma gangrenosum and antiphospholipid syndrome. Investigations revealed anaemia (haemoglobin **94 g/L** [9.4 g/dL]), elevated inflammatory markers (ESR **56 mm/h**, CRP **13 mg/L**), marked hypergammaglobulinaemia (IgG **>80 g/L** [>8,000 mg/dL]), ANA positivity (1:40 nucleolar) and anti-SSA positivity. Anti-dsDNA, ANCA, antiphospholipid antibodies and thrombophilia work-up were negative, while ADA2 enzyme activity was normal. The constellation of childhood-onset refractory ulcers, immune dysregulation and developmental delay prompted genetic evaluation. Whole-exome sequencing identified a **likely pathogenic homozygous deletion involving exons 10–11 of the *PEPD* gene** (chr19:g.(33401870_33411671)_(33413644_33462994)del), confirming prolidase deficiency and establishing the final diagnosis.

**Discussion:**

Prolidase deficiency is a rare autosomal recessive disorder caused by mutations in the **PEPD** gene, leading to impaired collagen degradation and defective connective tissue repair. Beyond its metabolic basis, it is increasingly recognised as an inborn error of immunity because of its association with immune dysregulation, recurrent infections, hypergammaglobulinaemia and autoimmune manifestations. Chronic painful lower-limb ulcers are the hallmark clinical feature and often represent the earliest manifestation. However, the rarity of the disease and its heterogeneous presentation frequently result in delayed diagnosis and prolonged exposure to ineffective immunosuppressive therapies.

Our patient posed a significant diagnostic challenge because the clinical presentation closely resembled inflammatory and vasculitic disorders encountered in rheumatology. Sequential skin biopsies suggested pyoderma gangrenosum and later livedoid vasculopathy, while positive ANA and anti-SSA antibodies further raised the possibility of connective tissue disease. Nevertheless, the absence of definitive systemic features, persistently refractory disease despite immunosuppressive therapy, marked hypergammaglobulinaemia, childhood onset and developmental delay were important clues indicating an alternative diagnosis. These atypical features prompted reconsideration of the initial diagnosis and subsequent genetic evaluation, which confirmed prolidase deficiency. This case highlights the importance of maintaining a broad differential diagnosis in young patients with chronic ulcerative dermatoses and immune dysregulation. Early recognition of monogenic disorders is essential to avoid unnecessary immunosuppression, provide accurate genetic counselling and optimise long-term multidisciplinary management.

**Key learning points:**

• This case emphasises several important learning points for rheumatologists. Childhood-onset, recurrent, treatment-refractory ulcerative skin lesions should prompt consideration of monogenic disorders in addition to autoimmune diseases and vasculitis. Although pyoderma gangrenosum, livedoid vasculopathy, cutaneous polyarteritis nodosa and connective tissue disease-associated vasculitis are common differentials, the presence of atypical features should encourage diagnostic reconsideration rather than escalation of immunosuppressive therapy alone. In our patient, developmental delay, marked hypergammaglobulinaemia, autoantibody positivity without a defined connective tissue disease, and failure to respond adequately to multiple immunosuppressive agents were key clues suggesting an inherited disorder.

• Prolidase deficiency should be recognised as an important rheumatological mimic because it may present with chronic ulcers, lupus-like autoimmunity, hypergammaglobulinaemia and vasculitis-like manifestations. Histopathological findings may also vary over time, further complicating the diagnostic process. This case reinforces the value of integrating clinical history, laboratory findings and genetic testing when conventional diagnoses fail to explain the overall clinical picture. Early molecular diagnosis not only establishes the correct diagnosis but also prevents prolonged ineffective treatment, enables appropriate surveillance for associated complications, facilitates genetic counselling and improves patient care through a multidisciplinary approach. Rheumatologists should maintain a high index of suspicion for inherited immune dysregulation syndromes when evaluating young patients with chronic ulcerative dermatoses and atypical autoimmune features.

